# Integrated epigenomic analyses of enhancer as well as promoter regions in gastric cancer

**DOI:** 10.18632/oncotarget.8239

**Published:** 2016-03-21

**Authors:** Su-Jin Baek, Mirang Kim, Dong-Hyuck Bae, Jeong-Hwan Kim, Hee-Jin Kim, Myoung-Eun Han, Sae-Ock Oh, Yong Sung Kim, Seon-Young Kim

**Affiliations:** ^1^ Department of Functional Genomics, University of Science and Technology, Daejeon, Republic of Korea; ^2^ Genomic Structure Research Center, Korea Research Institute of Bioscience and Biotechnology, Daejeon, Republic of Korea; ^3^ Epigenome Research Center, Korea Research Institute of Bioscience and Biotechnology, Daejeon, Republic of Korea; ^4^ Departments of Anatomy and Surgery, School of Medicine, Pusan National University, Busan, Republic of Korea

**Keywords:** gastric cancer, DNA methylation, enhancer, promoter, lncRNAs

## Abstract

Abnormal DNA methylation is an epigenetic mechanism that promotes gastric carcinogenesis. While the abnormal methylation at promoter regions has been characterized for many genes, the function of DNA methylation marks at distal regulatory regions in gastric cancer remains poorly described. Here, we performed RNA-seq, MBD-seq, and H3K27ac ChIP-seq on gastric tissues and cell lines to understand the epigenetic changes in the distal as well as the proximal regulatory regions. In total, 257,651 significant DMRs (Differentially methylated regions) were identified in gastric cancer, and the majority of these DMRs were located in the intergenic, intronic, and non-coding RNA regions. We identified the aberrant expression of many genes and lncRNAs due to changes in DNA methylation. Furthermore, we profiled the molecular subtype-specific methylation patterns in gastric cancer to characterize subtype-specific regulators that undergo DNA methylation changes. Our findings provide insights for understanding methylation changes at distal regulatory regions and reveal novel epigenetic targets in gastric cancer.

## INTRODUCTION

Gastric cancer (GC) is the fifth leading cause of cancer incidence and the third leading cause of cancer-related death after lung and liver cancer [1]. Multiple factors including molecular, genetic, and epigenetic changes as well as environmental factors (i.e., viral and bacterial infection) have been associated with the formation and progression of GC.

Cancer epigenetics comprise various fields, including DNA methylation, histone modifications, nucleosome positioning, and non-coding RNA. Among them, DNA methylation has long been studied as one of the important players during carcinogenesis, and promoter hypermethylation of several tumor suppressor genes (e.g., *p16*, *MLH1*, and *APC*) has been established as one of the key events. However, recent technological advances in genomic and epigenomic analyses have shown that distal regulatory regions such as enhancers are as important as proximal promoters in normal development and differentiation, and abnormal changes in the distal regulatory regions are associated with many diseases, including cancer. For example, Ziller et al. suggested that DNA methylation at enhancers is associated with key lineage specific regulators [2], and Aran et al. showed that methylation changes at distal regulatory sites were associated with gene expression in 58 cell types [3]. Additionally, long non-coding RNAs (lncRNAs) have drawn much interest as one of the important regulators during carcinogenesis and as potential biomarkers for early detection and prognosis and as predictive markers in multiple cancers [4].

Despite the growing interest in epigenomic changes at distal regulatory regions such as enhancers and lncRNAs, there have been few studies on the role of distal regulatory regions in GC. A few studies on DNA methylation in GC were performed with array-based DNA methylation profiling methods such as HumanMethylation27 and HumanMethylation450 BeadChip, with a focus on the proximal regulatory regions [5, 6]. To understand important epigenomic changes at the distal as well as proximal regulatory regions in GC compared with normal, we performed genome-wide methylation profiling using methyl-CpG binding domain sequencing (MBD-seq), H3K27ac ChIP-seq, and RNA-seq and performed integrated analyses of the epigenomic and transcriptomic data sets. Our results showed that the distal as well as the proximal regulatory regions are important during gastric carcinogenesis and should be studied further. Also, as recent TCGA work has shown that there are four distinct subtypes of GC showing different molecular patterns, we also investigated whether DNA methylation patterns change in a subtype-specific manner.

## RESULTS

### Genome-wide methylation profiling in GC

**Figure 1:**
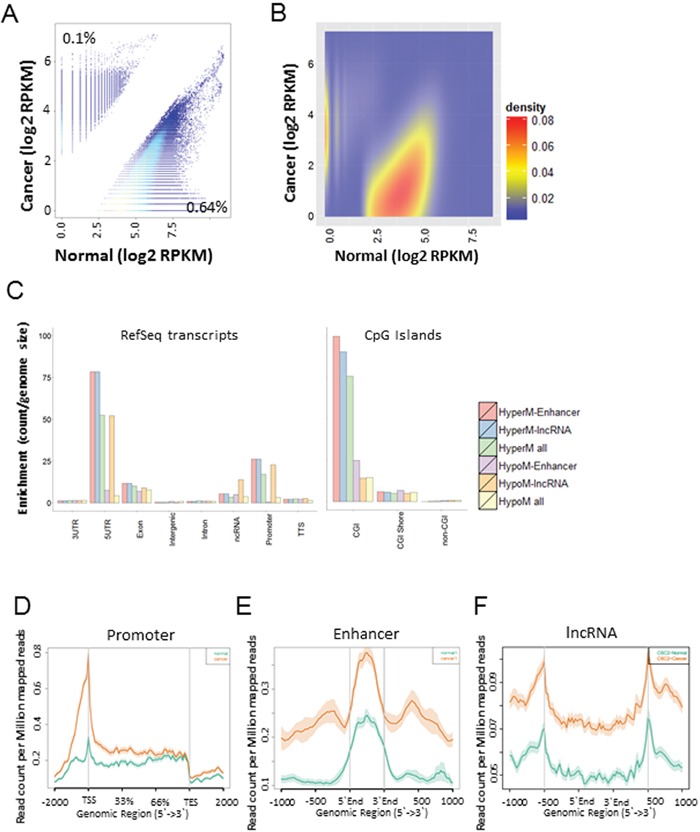
Genome-wide identification of differentially methylated regions (DMRs) in GC compared with normal tissues **A.** A scatter plot of differentially methylated regions in GC (|fc| > =2, and p-value <= 0.05). **B.** A heat scatter plot of differentially methylated regions. **C.** Distribution of DMRs across various genomic features. **D, E, F.** MBD methylation levels at the promoter (D), enhancer (E), and non-coding regions (F) in GC compared with normal. Orange color represents cancer, and green color represents normal tissues.

We profiled DNA methylomes using five GCs and two normal tissues to identify differentially methylated regions (DMRs) between the two groups (Supplementary Table S1). Approximately 0.1% (36,145 bins) of the hyper-methylated and 0.64% (221,506 bins) of the hypo-methylated DMRs (|fc| >= 2 and P-value < 0.05) were selected from a total of 34,496,773 bins (Figure 1A and 1B, Supplementary Data S1). On the whole, hypo-methylated regions were observed more frequently than hyper-methylated regions in GC, but hyper-methylated regions were more frequently observed in the 5′UTR, CGI and promoter areas than other regions (Figure 1C). Especially, lncRNAs in the 5′UTR and promoter regions (Gencode v. 19) were enriched with hypo-methylated DMR regions (Figure 1C). Most of the DMRs within the promoter, enhancer, and lncRNA regions were hyper-methylated in GC compared with the normal controls (Figure 1D, 1E, and 1F).

A few recent works showed that specific repetitive sequences (i.e. LINE-1) residing within some proto-oncogenes lead to the increased expression of oncogenes such as MET in colorectal cancer [7-9]. To identify over-expressed genes by hypomethylation at repetitive element in GC, we first selected 0.56% (3,128 regions) hypo-methylated repeat regions from a total of 5,298,130 repeat regions (University of California, Santa Cruz). Then the expression level of the closest gene was assessed for each hypo-methylated repeat. As a result, 574 over-expressed genes were chosen as candidates for hypomethylation at repeated elements (Supplementary Data S2). Among them, several genes including MCF21, FGFR3, RARA, MKRN2, AKAP13, VAV1, DACH1, and ZNF521 are known as oncogenes.

### Detection of epigenetically regulated genes due to methylation at promoters as well as enhancers

**Figure 2:**
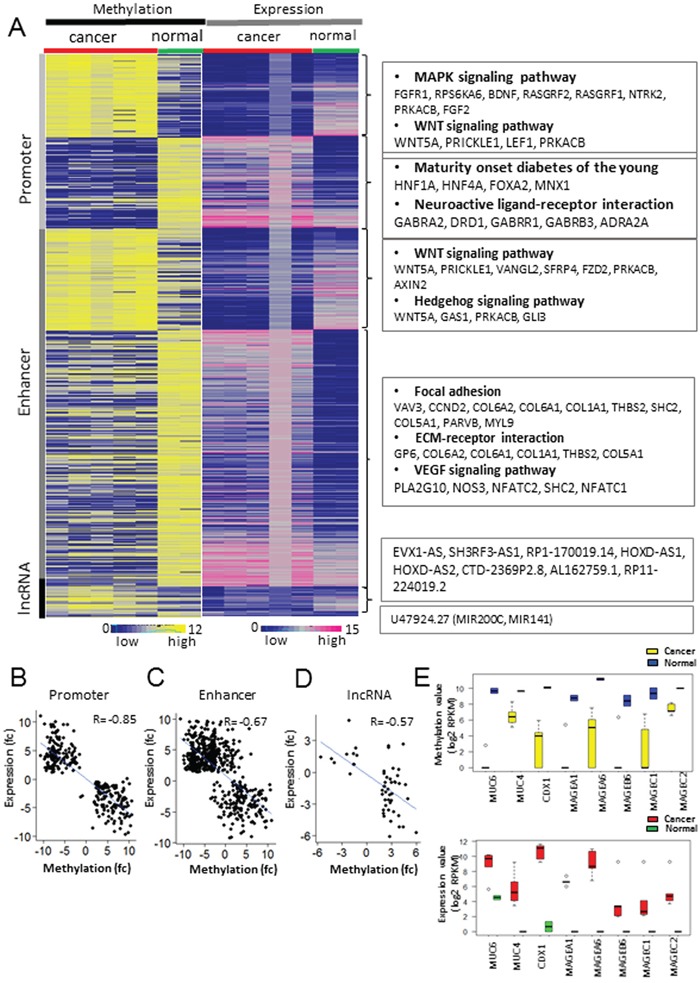
Integrated analysis of methylome and transcriptiome profiling in GC **A.** Matched heat map of gene expression and methylation profiling. **B, C, D.** Inverse correlation between methylation values of three regulatory regions and mRNA abundance. The scatter plot shows the correlation of gene expression and methylation at the promoter (B), enhancer (C), and non-coding regions (D). The blue line represents a linear regression. **E.** The methylation (upper) and expression (bottom) levels of hypo-methylated and up-regulated genes in GC compared with normal controls. The yellow and red box represents cancer samples, and the blue and green represents normal samples.

To identify functionally relevant DMRs, mRNA-seq data were utilized for finding a strong negative correlation between gene expression and DNA methylation at the promoter, enhancer, and lncRNA regions (Figure 2A). In promoter regions, 140 hyper and 154 hypo-methylated genes were identified as having significant negative correlation with the expression of their corresponding genes (Figure 2B and Supplementary Data S3). Functional annotation of these genes showed that the under-expressed genes with promoter hypermethylation were enriched in GO terms such as ‘MAPK signaling pathway’ (P-value: 0.0026), ‘Basal cell carcinoma’ (P-value: 0.0071), and ‘Hedgehog signaling pathway’ (P-value: 0.0075) (Figure 2A and Supplementary Table S2). The over-expressed genes with promoter hypomethylation were enriched in terms such as ‘maturity onset diabetes of the young (P-value: 5.68E-04)’ and ‘neuroactive ligand-receptor interactions (P-value: 0.08)’ (Figure 2A and Supplementary Table S3).

To investigate the functional significance of epigenetic changes at the distal regulatory regions in GC, we examined DNase-seq data (Supplementary Table S4) in 11 gastric tissues and H3K27ac ChIP-seq data (cell lines and tissue samples for ChIP-seq are listed in Materials and Methods) in 7 GC cell lines. We defined 134,322 enhancers in gastric tissue by intersecting 227,413 peaks from DNase-seq and 363,256 peaks from H3K27ac ChIP-seq for gastric-specific active enhancers. Then, the expression level of the closest gene was assessed for each enhancer. As a result, 606 genes were identified to have a strong negative correlation between gene expression and the DNA methylation of their enhancer regions (Figure 2C and Supplementary Data S4). Among them, 433 genes over-expressed due to enhancer hypo-methylation were enriched in GO terms such as ‘focal adhesion’ (*VAV3, CCND2, COL6A2, COL6A1*, and *THBS2;* P-value: 0.030) and ‘ECM-receptor interaction’ (*GP6, COL6A2, COL6A1, COL1S1, THBS2*, and *COL5A1*; P-value: 0.0357) (Figure 2A and Supplementary Table S5). Furthermore, motif analysis using the Homer tool revealed that motifs of the TEAD family genes such as TEAD1, TEAD2, and TEAD4 were enriched at the hypo-methylated enhancer regions (Supplementary Figure S1A) [10]. As de-methylation at enhancers is often associated with the binding of transcription factors during carcinogenesis [11, 12], we investigated whether those hypo-methylated enhancers were bound by *TEAD4* by analyzing the previously published *TEAD4* ChIP-seq data in two gastric cancer cell lines [13]. From the ChIP-seq study, 483 peaks (247 genes) from the MKN28 cell line and 232 peaks (102 genes) from the SNU216 cell line were found to be *TEAD4*-enriched and to contain hypo-methylated enhancer regions, among which 86 genes were common between the two cell lines (Supplementary Figure S1B). Enrichment analysis with the common 86 genes revealed that terms such as ‘cell adhesion molecules’ (P-value: 5.50E-03) and ‘pathway in cancer’ (P-value: 4.80E-02) were highly enriched (Supplementary Table S6) [14, 15]. Among these, the expression of *THBS2* and *CLDN15* showed a significant negative correlation with the methylation levels of *TEAD4*-enriched enhancers in GC compared with normal tissues (|fc| >= 2 and P-value < 0.05) (Supplementary Figure S1C, Supplementary Figure S2A and S2D). We validated the methylation and expression levels of THBS2 using TCGA data. *THBS2* was significantly over-expressed in 212 GCs compared with 28 normal samples from the TCGA cohorts (RNA-seq; Supplementary Figure S2B). We also investigated the methylation changes at the intergenic enhancer region of *THBS2* from the TCGA HumanMethylation 450 BeadChIP data and found that the *THBS2* enhancer region was significantly hypo-methylated in GC compared with normal tissues (Supplementary Figure S2C).

To investigate the potential of epigenetic change at gastric specific enhancers [16], we analyzed the recently published epigenome data from Roadmap Epigenomics Project (http://www.roadmapepigenomics.org/) including a few gastric samples (Supplementary Table S7). Among 216,695 gastric-specific enhancers, 7,826 (3.61%) were hyper-methylated, and 28,141 (12.99%) were hypo-methylated (Supplementary Data S5). In total, 4,911 genes were identified as closest gene at epigenetically changed enhancer regions. Among them, several genes were related in cancer pathway such as EGFR, RARA, FGF2, FGFR1, FGFR2, FGFR3, KIT, and CCND1.

### Epigenetically regulated lncRNAs

LncRNAs, non-protein coding transcripts longer than 200 nucleotides, have emerged as one of the important players during gastric carcinogenesis, and many lncRNAs have been shown to be aberrantly expressed in GC. Hence, we investigated the role of DNA methylation in the aberrant expression of lncRNAs in GC. Among 20,019 lncRNAs (Gencode v. 19), 1,497 (7.47%) were hyper-methylated, and 4,027 (20.21%) were hypo-methylated (Supplementary Figure S3A). Among them, 1,130 (5.64%) hyper-methylated and 2,139 (10.68%) hypo-methylated regions overlapped with enhancer peaks defined by the DNase-seq and H3K27ac ChIP-seq data (Supplementary Figure S3A). Combined analyses of the RNA-seq and MBD-seq data revealed 41 lncRNAs under-expressed due to hypermethylation and 12 lncRNAs over-expressed due to hypomethylation (Figure 2A; Supplementary Data S6). Of these lnc-RNAs, the expression of *MALAT1*, *EVX-AS*, *SH3RF3-AS*, *RP-17-19.14* (reverse strand of *HOXA11-AS*), *HOXD-AS1*, and *HOXD-AS2* showed a significant negative correlation with the DNA methylation levels at the promoters of the lncRNAs (Figure 2D). Additionally, the *U47924.27* (Gencode identification) locus containing *MIR200C* and *MIR141* was found to be hypo-methylated at its promoter and over-expressed in GC (Figure 2A; Supplementary Figure S3B). *MALAT1* is known to be frequently over-expressed in human malignancies including GC [17], and Supplementary Figure S4A shows that *MALAT1* was significantly over-expressed in GC due to promoter hypo-methylation. We validated the methylation and expression levels of *MALAT1* using the TCGA cohort. Two of the eight probes located in the promoter of *MALAT1* were significantly hypo-methylated in GC according to data from the TCGA (HumanMethylation450k beadchip) (Supplementary Figure S4B). Additionally, the expression level of *MALAT1* in GC using the 240 RNA-sequencing data from the TCGA cohorts was assessed and showed that *MALAT1* was significantly over-expressed in GC compared with normal tissues (Supplementary Figure S4C). To verify whether 5-Aza-dC influences *MALAT1* expression, we treated four gastric cancer cell lines with 5-Aza-dC. The expression of *MALAT1* in the four gastric cancer cell lines (SNU620, SNU005, SNU016, and AGS) was increased by 5-Aza-dC treatment (Supplementary Figure S4D). This result indicates that changes in methylation at *MALAT1* promoter may contribute to the over-expression of *MALAT1* in GC.

### Over-expression of *HNF4A* due to promoter hypo-methylation in GC compared with normal

**Figure 3:**
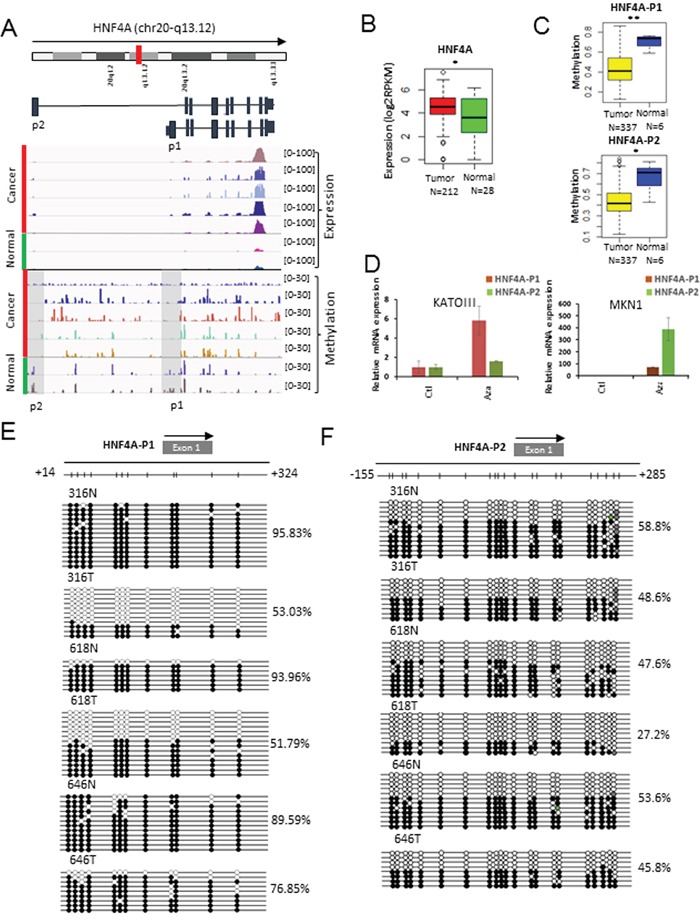
Methylation profiling of *HNF4A* as a target for promoter hypo-methylation **A.** Screenshot of the IGV browser shows the promoter of *HNF4A.* Display tracks include the abundance of promoter methylation and expression. **B.** The expression levels of *HNF4A* in 240 gastric tissues from the TCGA cohorts (mRNA-seq). The red box represents cancer samples and the green box represents normal samples (*: p-value < 0.05; **: p-value < 0.005; ***: p-value < 0.0005). **C.** The methylation levels of HNF4A-P1 (Top) and HNF4A-P2 (Bottom) in GC and normal tissues from the HumanMethylation450k BeadChip array. The yellow box represents cancer samples and the blue box represents normal samples (*: p-value < 0.05; **: p-value < 0.005; ***: p-value < 0.0005). **D.** *HNF4A* over-expression by 5-Aza-dC in gastric cancer cell lines. Quantitative RT-PCR was performed using P1 and P2 specific primers for HNF4A. The red bar represents HNF4A-P1 and the green bar represents HNF4A-P2. **E.** Bisulfite sequencing result of CpG sites (n=12) in the HNF4A-P1 promoter region. **F.** Bisulfite sequencing result of CpG sites (n=21) in the HNF4A-P2 promoter region.

To identify novel epigenetically altered oncogenes, we focused on genes that are over-expressed due to promoter hypo-methylation. One of the epigenetically altered genes was *HNF4A*, known as a key member of the nuclear receptor subfamily of ligand-dependent receptors. Chang et al. recently showed that *HNF4A* is a novel therapeutic target that links AMPK to WNT signaling in early-stage gastric cancer [18]. Interestingly, two distinct promoters (HNF4A-P1 and HNF4A-P2) are known to increase the expression of *HNF4A* in some cancers. Thus, we examined the methylation of the two promoters and the expression level of *HNF4A* in GC tissues and cell lines (Figure 3A). *HNF4A* was significantly over-expressed in 212 GCs compared with 28 normal samples from the TCGA cohorts (Figure 3B). Additionally, the promoter methylation levels of HNF4A-P1 and HNF4A-P2 were confirmed using the HumanMethylation450k BeadChip array from the TCGA cohorts (Figure 3C) and were inversely correlated with their expression in GCs compared with normal controls. However, we found that both HNF4A-P1 and HNF-P2 were coordinately methylated in GC (Figure 3C). Hypomethylation of the *HNF4A* promoter region was independently validated using the MENT database (Supplementary Figure S5A) [19].

To verify whether 5-Aza-dC, an inhibitor of the DNMT enzymes, influences *HNF4A* expression, we treated two gastric cell lines with 5-Aza-dC. We first performed quantitative RT-PCR to measure the expression levels of *HNF4A*-P1 and *HNF4A*-P2 in diverse gastric cancer cell lines and selected two cell lines (KATO-III and MNK1), which expressed HNF4A-P1 and HNF4A-P2 at low levels (Supplementary Figure S5B and S5C). The expression of *HNF4A* in KATO-III and MKN1 was augmented by 5-Aza-dC treatment (Figure 3D). This result indicates that changes in methylation at *HNF4A* promoters may contribute to the over-expression of *HNF4A* during the development of GC. We also performed bisulfite sequencing to measure the methylation levels of HNF4A-P1 (Figure 3E; P-value: 0.01) and HNF4A-P2 (Figure 3F; P-value: 0.16) in three pairs of GC and adjacent normal tissues. Both promoters of *HNF4A* were hypo-methylated in the GC tissues compared with the normal controls.

### GC molecular subtype specific DMRs

**Figure 4:**
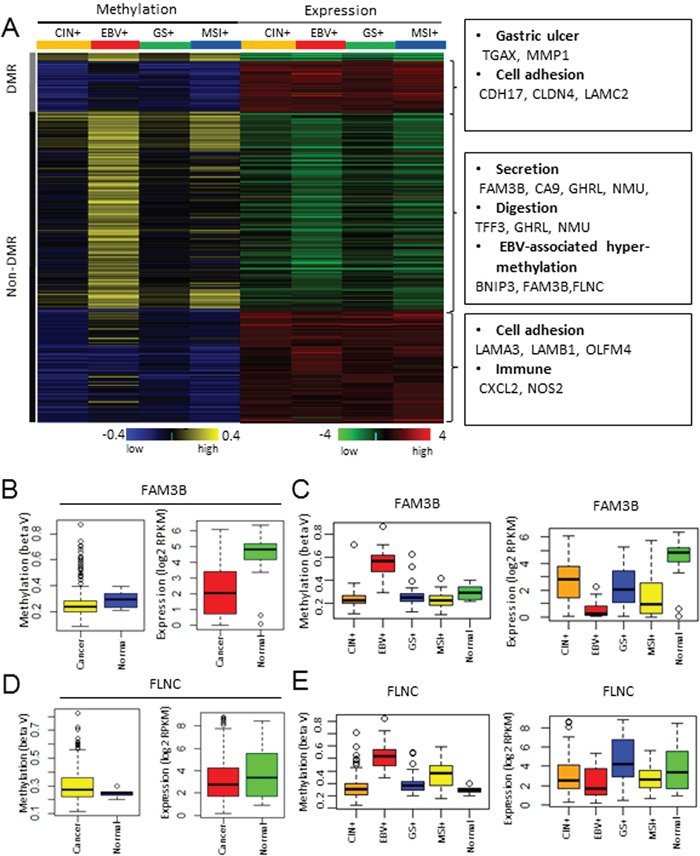
Molecular subtype-specific methylation and expression profiling in GC **A.** Methylation and expression profiling of subtype-specific methylation targets. **B.** *FAM3B* promoter methylation (left) and mRNA expression (right) levels in GC and normal tissues from the TCGA cohorts. The yellow and red box indicates cancer samples and the blue and green box indicates normal samples. **C.** *FAM3B* promoter methylation (left) and mRNA expression (right) levels in the normal tissues, and the CIN+, EBV+, GS+, and MSI+ gastric tumor subtypes. **D.** *FLNC* promoter methylation (left) and mRNA expression (right) levels in GC and normal tissues from the TCGA cohorts. The yellow and red box indicates cancer samples and the blue and green box indicates normal samples. **E.** *FLNC* promoter methylation (left) and mRNA expression (right) levels in the normal tissues, and the CIN+, EBV+, GS+, and MSI+ gastric tumor subtypes.

A recent TCGA study on gastric cancer classified GC into four distinct molecular subtypes: CIN+, EBV+, GS+, and MSI+ [20]. Among them, the EBV+ and MSI+ subtypes are characterized by extensive DNA methylation. Subtype-specific DMRs were predicted using the TCGA stomach cancer (STAD) methylation and expression data, which comprise HumanMethylation450K for CIN+ (n=122), EBV+ (n=25), GS+ (n=52), MSI+ (n=49); six normal samples and RNA-seq data for CIN+ (n=101), EBV+ (n=19), GS+ (n=41), and MSI+ (n=41) gastric cancer; and 28 normal samples (Supplementary Data S7). Student's t-test was used to compare the samples from each subtype and normal group, and 379 hyper-methylated and under-expressed genes and 309 hypo-methylated and over-expressed genes were selected (Figure 4A; Supplementary Data S8). Among them, 355 genes were under-expressed due to subtype-specific hyper-methylation. Functional enrichment analysis revealed the enrichment of GO categories such as secretion (*FAM3B*, *CA9*, *GHRL*, *NMU*), digestion (*TFF3*, *GHRL*, *NMU*) and EBV-associated hyper-methylation (*BNIP3*, *FAM3B*, *FLNC*) (Figure 4A). Two examples of EBV+ specific genes, *FAM3B* and *FLNC*, were shown to exemplify the importance of subtype-specific analysis.

For *FAM3B*, the difference in methylation between the cancer and normal groups was insignificant, although a subset of samples showed a distinct pattern of hyper-methylation (Figure 4B, left panel). On the contrary, the difference in expression between the cancer and normal groups was significant (Figure 4B, right panel). Subtype-specific analysis revealed that only the EBV+ subtype GCs showed hyper-methylation of *FAM3B* (Figure 4C, left panel), while the other subtype GCs had similar methylation patterns to the normal group. The EBV+ subtype GCs showed the most decreased expression of *FAM3B* (Figure 4C, right panel), but the other subtype GCs also showed modest under-expression of *FAM3B* compared with the normal groups, suggesting that the mechanism of *FAM3B* under-expression is distinct from DNA methylation. Thus, subtype specific analysis clearly showed that the EBV+ subtype and the other subtypes had a distinct mechanism of *FAM3B* under-expression in GC. For *FLNC*, differences in both methylation and expression between the GC and normal groups were insignificant (Figure 4D). However, subtype-specific analysis again revealed that *FLNC* was under-expressed due to hyper-methylation in the EBV+ subtype alone (Figure 4E).

## DISCUSSION

Several groups have previously reported genome-wide DNA methylation changes in GC using chip-based methods or MIRA-seq technology. Zouridis et al. generated methylation profiles of 240 GCs and 94 matched normal samples using the HumanMethylation27k array [21]. The TCGA consortium recently reported the methylation profiling of 295 GCs using the HumanMethylation450k array [20]. Park et al. used MIRA-seq to characterize genome-wide methylation patterns at CpG rich regions and repeat regions using three GC and matched normal samples [22]. Muratani et al. reported chromatin alteration at the promoters and predicted enhancers focusing on histone modifications in primary gastric cancer in multiple ChIP-seq datasets [23]. However, the above-mentioned studies focused mainly on the proximal regions using array platforms which does not cover the potentially important distal regulatory regions, due to the limitation of the array probe design. In contrast, our study assessed genome-wide DNA methylation changes not only at the proximal regions but also at the distal regulatory elements in GC.

By combining MBD-seq with H3K27ac ChIP-seq and DNase-seq, we identified a large number of gastric enhancers with DNA methylation changes. We further showed that hypo-methylated-enhancer sites were enriched with the *TEAD4* motif, some of which were bound by *TEAD4*, leading to increases in the expression of their respective genes (Supplementary Figure S1C). *THBS2* has been implicated as a modulator of cell surface properties that are involved in cell adhesion and migration [24, 25]. The expression of *THBS2* was significantly increased in colon, esophagus, lung, and stomach cancers as analyzed using the GENT database (Supplementary Figure S6A) [26]. Notably, we found that *THBS2* was over-expressed due to de-methylation at its enhancer containing *TEAD4* binding sites (Supplementary Figure S2A). We also investigated the methylation changes at the intergenic enhancer region of *THBS2* in various cancers from the TCGA data (HumanMethylation450 BeadChIP) and found that the enhancer of *THBS2* was significantly hypo-methylated in colon and liver cancers (Supplementary Figure S6B and S6D) but not in kidney and pancreatic cancers (Supplementary Figure S6C and S6E). These results indicate that DNA methylation changes at the distal enhancers occur in a tissue-specific manner.

Integrated analysis of promoter methylation and expression revealed many potential epi-driver genes including *MUC6*, *MUC4*, *CDX1*, *HNF4A*, MAGE family genes (*MAGEA1*, *MAGEA6*, *MAGEC1*, and *MAGEC2*), which were hypo-methylated at their promoters and were over-expressed (Figure 2E). Further studies are needed to evaluate whether the above mentioned genes have oncogenic activity. Among them, expression of *HNF4A* is increased by alternative promoters in several cancers such as liver, gastric, and colorectal cancers [27]. Hence, we investigated whether the two promoters of HNF4A (HNF4A-P1 and HNF4A-P2) were differentially methylated. But, our result indicates that HNF4A expression was increased by simultaneous de-methylation of the two promoters (Figure 3). Similarly, both promoters of *HNF4A* were significantly hypo-methylated in liver and colon cancers (Supplementary Figure S7A, and S7B). On the other hand, in pancreatic cancer, only P1-promoter was significantly hypo-methylated without P2-promoter hypo-methylation (Supplementary Figure S7C). Thus, we conclude that DNA methylation may play an important role by switching the active promoter of *HNF4A* in a tissue-specific manner.

As another example of epigenetic regulation, we analyzed methylation changes at the promoter regions of lncRNAs and identified putative lncRNAs such as *MALAT1*, *HOXD-AS1*, *HOXD-AS2*, and *EBV1-AS* (Figure 2A). Over-expression of *MALAT1* is frequently detected in human malignancies including GC, and it has been suggested as an oncogene [28-30]. We discovered that promoter de-methylation of MALAT1 could be a major mechanism for its over-expression in GC (Supplementary Figure S4).

We characterized subtype-specific methylation changes in GC for each of the four subtypes (CIN+, EBV+, GS+, and MSI+) by analyzing the HumanMethylation450k data. As previously reported [20], the EBV+ subtype was overwhelmingly hyper-methylated compared with the other molecular subtypes (Figure 4A). Several genes including *FAM3B* and *FLNC* were identified as hyper-methylated in the EBV+ subtype by subtype specific-analysis (Figure 4B, 4C, 4D, and 4E) [31, 32].

In conclusion, we highlight the importance of DNA methylation marks at the distal regulatory regions and lncRNAs as well as the proximal promoters in GC. Many DMRs were identified at the distal regulatory regions in GC and may have functional roles in the epigenetic regulation of gene expression.

## MATERIALS AND METHODS

### Cell lines and tissue samples

Seven gastric cancer cell lines (AGS, MKN1, MKN45, SNU719, SNU016, KATOIII, and SNU638) were obtained from the Korean Cell Line Bank (http://cellbank.snu.ac.kr/english/index.php) and cultured in RPMI 1640 medium supplemented with 10% fetal bovine serum and 1% antibiotic-antimycotic solution (Invitrogen, Carlsbad, CA). Paired gastric tumor and adjacent non-cancerous gastric mucosae samples were collected from the Pusan National University Yangsan Hospital. The study protocol was approved by the institutional review board of the hospital.

### MBD-seq and data analysis

MBD2-immunoprecipitated chromatin fragments were collected and 200~300-bp size-selected genomic libraries were prepared from DNA samples using the TruSeq ChIP Sample Prep Kit (Illumina). The libraries were multiplexed and sequenced on the Illumina platforms (GA IIx or Hiseq-2000). After sequencing, single-end reads (51 bp or 76 bp) were aligned against the human reference genome 19 with the BWA aligner [33]. The MEDIPS R package (v. 1.18.0) was used for the analysis of MBD-seq data and DMR identification [34]. The Homer (v.4.7) software was used for the annotation of peaks and assessment of the distribution of methylation peaks across genomic features [10]. Genomic features were classified into six regions: intergenic, 5 UTR, 3 UTR, promoter, CDS, and intron, based on the UCSC genome annotation information. For the locations related to a CGI (CpG island), we used three groups: within CGI, in CGI shore (2 kb region from the CGI), and non-CGI. All the MBD sequencing data were submitted to the public repository (GEO Accession Number: GSE46595). Bam files were available at http://mgrc.kribb.re.kr/KRIBB/BAM/MBD-seq/MBD-seq.html.

### RNA-seq and data analysis

The RNA sequencing library was prepared using the TruSeq RNA Sample Prep Kit (Illumina, San Diego, CA, USA) and sequenced using Genome Analyzer IIx (Illumina) or Hiseq-2000 (Illumina) to generate 76 or 101-bp paired end reads. The sequenced reads were mapped to the human genome (hg19) using TopHat 2, and the gene expression levels (GRCh37; Gencode ver 19) were quantified with the HTSeq package [35, 36]. The edgeR package was used to select differentially expressed genes from the RNA-seq count data [37]. Meanwhile, the RPKM value- of each gene was floored to 1, and log2-transformed for further analysis (heatmap and correlation analysis). All RNA sequencing data were deposited in the public repository (GEO Accession Number: GSE46597). Bam files were available at http://mgrc.kribb.re.kr/KRIBB/BAM/RNA-seq/RNA-seq.html.

### ChIP-seq and data analysis

ChIP-sequencing was performed for seven gastric cancer cell lines. A protocol generated at the Myers laboratory (http://hudsonalpha.org/myers-lab/protocols) was used with minor modifications. First, seven gastric cancer cell lines were fixed using 1% formaldehyde, lysed and sonicated using a Bioruptor (Diagenode Inc.). Then, 20 μg of H3K27ac antibody was prebound to 100 μl Dynabeads Protein (Life Technologies). For ChIP-sequencing, 250-400 bp genomic libraries were generated from the input and chromatin immunoprecipitated DNA and sequenced using Genome Analyzer IIx or Hiseq-2000 to generate 51 bp or 101 bp single-end reads. The sequencing reads were also aligned to the reference sequence (hg 19) using the BWA software. The results generated by MACS (v.1.4.2) [38] were loaded into the IGV for visualization [39]. PeakAnnotator from Homer (v 4.7) was used to annotate each peak. All ChIP sequencing data were deposited in the public repository (GEO Accession Number: GSE75595). Bam files were available at http://mgrc.kribb.re.kr/KRIBB/BAM/ChIP-seq/ChIP-seq.html.

### Methylation analyses of distal regulatory regions

Promoter regions were defined as 2 kb upstream of TSS to 0.5 kb downstream of TSS. Enhancer regions were defined from H3K27ac ChIP-seq and DNase-seq peaks. First, H3K27ac ChIP-seq peaks were detected by MACS, and each peak from seven gastric cell lines was merged into a total of 363,256 peaks using mergedBed of bedtools (ver. 2.17.0) [40]. Second, DHS peaks were detected by Homer and each peak from 11 gastric tissues was merged into a total 227,413 peaks using mergedBed of bedtools. The intersectBed command in bedtools was used to determine overlapping regions between differentially methylated regions and DHS at active enhancer regions. The targets of enhancer were selected from their nearest promoters on the same chromosome. The correlation between the promoter DNA methylation and expression of each lncRNA was measured with Pearson's correlation coefficient in the R-package.

### Analysis of subtype-specific methylation

GC methylation and expression data were obtained from the TCGA (available at the https://tcga-data.nci.nih.gov/tcga/). Each sample was grouped into four distinct molecular subtypes as defined in [20]. Subtype-specific DMRs were identified by comparing tumors in each subtype and six normal samples. Significance was inferred using Student's t-test.

### Public data process

Processed DNA methylation (HumanMethylation450k) and expression (mRNA-sequencing) data of stomach adenocarcinomas (STAD) were downloaded from The Cancer Genome Atlas data portal (https://tcga-data.nci.nih.gov/tcga/). Clinical and molecular-subtype information was obtained from the TCGA project article [20]. The sample details are described in Supplementary Data S7. The DNase-seq data of fetal stomach were downloaded from the Gene Expression Omnibus database (GSE189727). The sample information of the DNase-seq data is described in Supplementary Table S4.

### Bisulfite sequencing

Genomic DNA (2 μg) from each sample was modified by sodium bisulfite using the EZ DNA methylation kit (Zymo Research), according to the manufacturer's instructions, and PCR amplified. The PCR products were cloned into pGEM-T Easy vector (Promega), and several clones were randomly chosen for sequencing. Bisulfite-modified DNA was amplified using primer sets designed to amplify sites +14 to +324 for HNF4A-P1 and -155 to +285 for HNF4A-P2 using MethPrimer (http://www.urogene.org/cgi-bin/methprimer/methprimer.cgi). Primer sequences used for HNF4A-P1 were 5′-GGTAGAGAGGGTATTGGGAGGAGGTA-3′ (forward) and 5′-CCCACCCCAAAATTAAATACCAAAA-3′ (reverse). The primer sequences used for HNF4A-P2 were 5′-TATTTTGGGTGATTAGAAGAATTAA-3′ (forward) and 5′-CCTCTACCCCAAAACTTCTC-3′ (reverse).

### 5-aza-2′-deoxycytidine

Gastric cancer cell lines (KATOIII, MKN1, SNU620, SNU005, SNU016, and AGS) were seeded in 100 mm dishes at a density of 1×10^6^ cells/dish, treated with 10 μM 5-Aza-dC (Sigma-Aldrich) every 24 h for 3 days and then harvested.

## SUPPLEMENTARY FIGURES AND TABLES



















## References

[1] Wang K, Liang Q, Li X, Tsoi H, Zhang J, Wang H, Go MY, Chiu PW, Ng EK, Sung JJ, Yu J (2015). MDGA2 is a novel tumour suppressor cooperating with DMAP1 in gastric cancer and is associated with disease outcome. Gut.

[2] Ziller MJ, Gu H, Muller F, Donaghey J, Tsai LT, Kohlbacher O, De Jager PL, Rosen ED, Bennett DA, Bernstein BE, Gnirke A, Meissner A (2013). Charting a dynamic DNA methylation landscape of the human genome. Nature.

[3] Aran D, Sabato S, Hellman A (2013). DNA methylation of distal regulatory sites characterizes dysregulation of cancer genes. Genome Biol.

[4] Kanda M, Kodera Y (2015). Recent advances in the molecular diagnostics of gastric cancer. World J Gastroenterol.

[5] Stirzaker C, Zotenko E, Song JZ, Qu W, Nair SS, Locke WJ, Stone A, Armstong NJ, Robinson MD, Dobrovic A, Avery-Kiejda KA, Peters KM, French JD, Stein S, Korbie DJ, Trau M (2015). Methylome sequencing in triple-negative breast cancer reveals distinct methylation clusters with prognostic value. Nat Commun.

[6] Jin SG, Kadam S, Pfeifer GP (2010). Examination of the specificity of DNA methylation profiling techniques towards 5-methylcytosine and 5-hydroxymethylcytosine. Nucleic Acids Res.

[7] Roman-Gomez J, Jimenez-Velasco A, Agirre X, Cervantes F, Sanchez J, Garate L, Barrios M, Castillejo JA, Navarro G, Colomer D, Prosper F, Heiniger A, Torres A (2005). Promoter hypomethylation of the LINE-1 retrotransposable elements activates sense/antisense transcription and marks the progression of chronic myeloid leukemia. Oncogene.

[8] Hur K, Cejas P, Feliu J, Moreno-Rubio J, Burgos E, Boland CR, Goel A (2014). Hypomethylation of long interspersed nuclear element-1 (LINE-1) leads to activation of proto-oncogenes in human colorectal cancer metastasis. Gut.

[9] Zhu C, Utsunomiya T, Ikemoto T, Yamada S, Morine Y, Imura S, Arakawa Y, Takasu C, Ishikawa D, Imoto I, Shimada M (2014). Hypomethylation of long interspersed nuclear element-1 (LINE-1) is associated with poor prognosis via activation of c-MET in hepatocellular carcinoma. Ann Surg Oncol.

[10] Heinz S, Benner C, Spann N, Bertolino E, Lin YC, Laslo P, Cheng JX, Murre C, Singh H, Glass CK (2010). Simple combinations of lineage-determining transcription factors prime cis-regulatory elements required for macrophage and B cell identities. Mol Cell.

[11] Kulis M, Merkel A, Heath S, Queiros AC, Schuyler RP, Castellano G, Beekman R, Raineri E, Esteve A, Clot G, Verdaguer-Dot N, Duran-Ferrer M, Russinol N, Vilarrasa-Blasi R, Ecker S, Pancaldi V (2015). Whole-genome fingerprint of the DNA methylome during human B cell differentiation. Nat Genet.

[12] Huang CZ, Yu T, Chen QK (2015). DNA Methylation Dynamics During Differentiation, Proliferation, and Tumorigenesis in the Intestinal Tract. Stem Cells Dev.

[13] Lim B, Park JL, Kim HJ, Park YK, Kim JH, Sohn HA, Noh SM, Song KS, Kim WH, Kim YS, Kim SY (2014). Integrative genomics analysis reveals the multilevel dysregulation and oncogenic characteristics of TEAD4 in gastric cancer. Carcinogenesis.

[14] Huang da W, Sherman BT, Lempicki RA (2009). Bioinformatics enrichment tools: paths toward the comprehensive functional analysis of large gene lists. Nucleic Acids Res.

[15] Huang da W, Sherman BT, Lempicki RA (2009). Systematic and integrative analysis of large gene lists using DAVID bioinformatics resources. Nat Protoc.

[16] Roadmap Epigenomics C, Kundaje A, Meuleman W, Ernst J, Bilenky M, Yen A, Heravi-Moussavi A, Kheradpour P, Zhang Z, Wang J, Ziller MJ, Amin V, Whitaker JW, Schultz MD, Ward LD, Sarkar A (2015). Integrative analysis of 111 reference human epigenomes. Nature.

[17] Wang J, Su L, Chen X, Li P, Cai Q, Yu B, Liu B, Wu W, Zhu Z (2014). MALAT1 promotes cell proliferation in gastric cancer by recruiting SF2/ASF. Biomed Pharmacother.

[18] Chang HR, Nam S, Kook MC, Kim KT, Liu X, Yao H, Jung HR, Lemos R, Seo HH, Park HS, Gim Y, Hong D, Huh I, Kim YW, Tan D, Liu CG (2016). HNF4alpha is a therapeutic target that links AMPK to WNT signalling in early-stage gastric cancer. Gut.

[19] Baek SJ, Yang S, Kang TW, Park SM, Kim YS, Kim SY (2013). MENT: methylation and expression database of normal and tumor tissues. Gene.

[20] Cancer Genome Atlas Research N (2014). Comprehensive molecular characterization of gastric adenocarcinoma. Nature.

[21] Zouridis H, Deng N, Ivanova T, Zhu Y, Wong B, Huang D, Wu YH, Wu Y, Tan IB, Liem N, Gopalakrishnan V, Luo Q, Wu J, Lee M, Yong WP, Goh LK (2012). Methylation subtypes and large-scale epigenetic alterations in gastric cancer. Sci Transl Med.

[22] Park JH, Park J, Choi JK, Lyu J, Bae MG, Lee YG, Bae JB, Park DY, Yang HK, Kim TY, Kim YJ (2011). Identification of DNA methylation changes associated with human gastric cancer. BMC Med Genomics.

[23] Muratani M, Deng N, Ooi WF, Lin SJ, Xing M, Xu C, Qamra A, Tay ST, Malik S, Wu J, Lee MH, Zhang S, Tan LL, Chua H, Wong WK, Ong HS (2014). Nanoscale chromatin profiling of gastric adenocarcinoma reveals cancer-associated cryptic promoters and somatically acquired regulatory elements. Nat Commun.

[24] Segal E, Sirlin CB, Ooi C, Adler AS, Gollub J, Chen X, Chan BK, Matcuk GR, Barry CT, Chang HY, Kuo MD (2007). Decoding global gene expression programs in liver cancer by noninvasive imaging. Nat Biotechnol.

[25] Yang S, Shin J, Park KH, Jeung HC, Rha SY, Noh SH, Yang WI, Chung HC (2007). Molecular basis of the differences between normal and tumor tissues of gastric cancer. Biochim Biophys Acta.

[26] Shin G, Kang TW, Yang S, Baek SJ, Jeong YS, Kim SY (2011). GENT: gene expression database of normal and tumor tissues. Cancer Inform.

[27] Tanaka T, Jiang S, Hotta H, Takano K, Iwanari H, Sumi K, Daigo K, Ohashi R, Sugai M, Ikegame C, Umezu H, Hirayama Y, Midorikawa Y, Hippo Y, Watanabe A, Uchiyama Y (2006). Dysregulated expression of P1 and P2 promoter-driven hepatocyte nuclear factor-4alpha in the pathogenesis of human cancer. J Pathol.

[28] Okugawa Y, Toiyama Y, Hur K, Toden S, Saigusa S, Tanaka K, Inoue Y, Mohri Y, Kusunoki M, Boland CR, Goel A (2014). Metastasis-associated long non-coding RNA drives gastric cancer development and promotes peritoneal metastasis. Carcinogenesis.

[29] Zhou X, Liu S, Cai G, Kong L, Zhang T, Ren Y, Wu Y, Mei M, Zhang L, Wang X (2015). Long Non Coding RNA MALAT1 Promotes Tumor Growth and Metastasis by inducing Epithelial-Mesenchymal Transition in Oral Squamous Cell Carcinoma. Sci Rep.

[30] Yoshimoto R, Mayeda A, Yoshida M, Nakagawa S (2016). MALAT1 long non-coding RNA in cancer. Biochim Biophys Acta.

[31] Liang Q, Yao X, Tang S, Zhang J, Yau TO, Li X, Tang CM, Kang W, Lung RW, Li JW, Chan TF, Xing R, Lu Y, Lo KW, Wong N, To KF (2014). Integrative identification of Epstein-Barr virus-associated mutations and epigenetic alterations in gastric cancer. Gastroenterology.

[32] Chang MS, Uozaki H, Chong JM, Ushiku T, Sakuma K, Ishikawa S, Hino R, Barua RR, Iwasaki Y, Arai K, Fujii H, Nagai H, Fukayama M (2006). CpG island methylation status in gastric carcinoma with and without infection of Epstein-Barr virus. Clin Cancer Res.

[33] Li H, Durbin R (2010). Fast and accurate long-read alignment with Burrows-Wheeler transform. Bioinformatics.

[34] Lienhard M, Grimm C, Morkel M, Herwig R, Chavez L (2014). MEDIPS: genome-wide differential coverage analysis of sequencing data derived from DNA enrichment experiments. Bioinformatics.

[35] Anders S, Pyl PT, Huber W (2015). HTSeq-a Python framework to work with high-throughput sequencing data. Bioinformatics.

[36] Kim D, Pertea G, Trapnell C, Pimentel H, Kelley R, Salzberg SL (2013). TopHat2: accurate alignment of transcriptomes in the presence of insertions, deletions and gene fusions. Genome Biol.

[37] Robinson MD, McCarthy DJ, Smyth GK (2010). edgeR: a Bioconductor package for differential expression analysis of digital gene expression data. Bioinformatics.

[38] Zhang Y, Liu T, Meyer CA, Eeckhoute J, Johnson DS, Bernstein BE, Nusbaum C, Myers RM, Brown M, Li W, Liu XS (2008). Model-based analysis of ChIP-Seq (MACS). Genome Biol.

[39] Thorvaldsdottir H, Robinson JT, Mesirov JP (2013). Integrative Genomics Viewer (IGV): high-performance genomics data visualization and exploration. Brief Bioinform.

[40] Quinlan AR, Hall IM (2010). BEDTools: a flexible suite of utilities for comparing genomic features. Bioinformatics.

