# Supplementary material for: Integrated epigenomic analyses of enhancer as well as promoter regions in gastric cancer
Source: Oncotarget. 2016 Mar 21;7(18):25620–31. doi: 10.18632/oncotarget.8239 (PMC5041931; doi:10.18632/oncotarget.8239)
Supplement: Supplementary file 1 [file oncotarget-07-25620-s001.pdf]

Integrated epigenomic analyses of enhancer as well as promoter regions in gastric cancer

SUPPLEMENTARY FIGURES AND TABLES

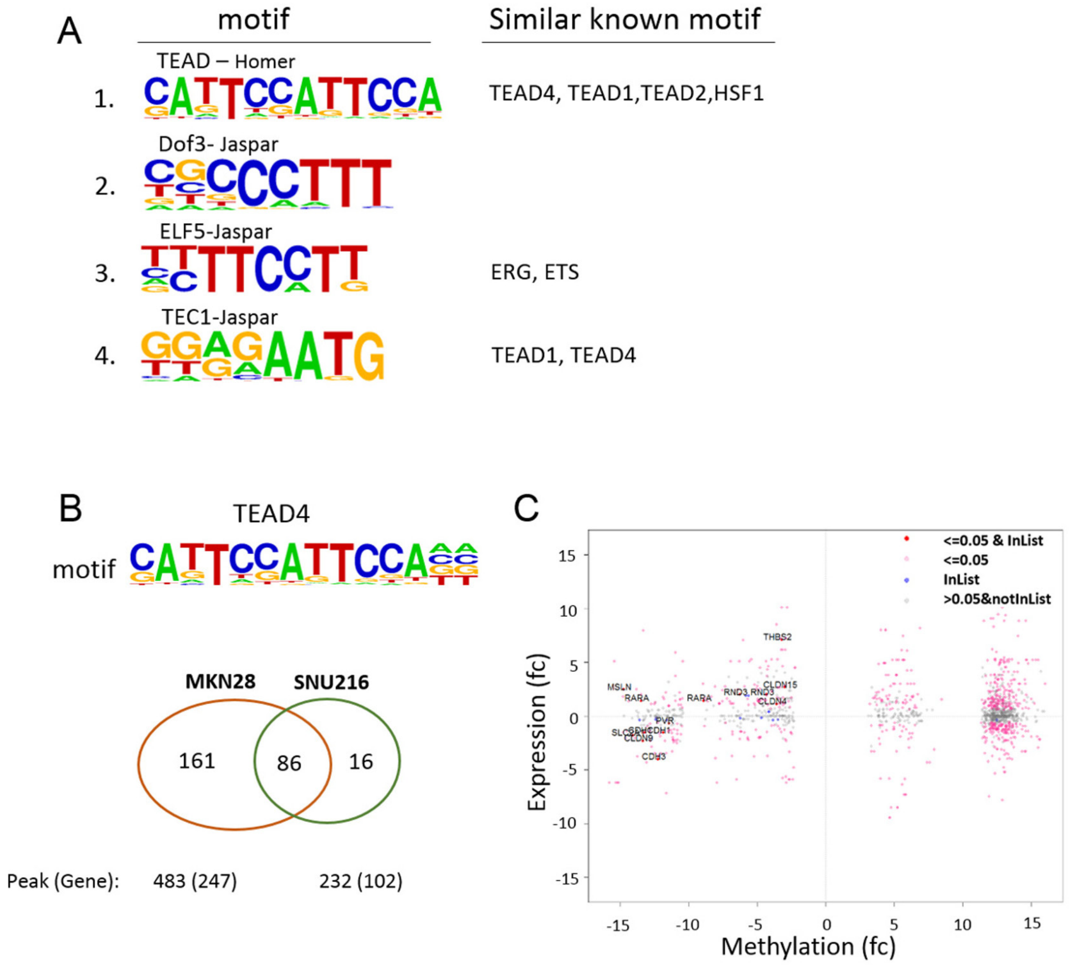

Supplementary Figure S1: Motif analysis of the hypo-methylated enhancer regions. A. Sequence motifs identified by de novo motif analysis of hypo-methylated enhancers. **B.** Closest gene of *TEAD4* enriched peaks with hypo-methylated enhancer in two gastric cell lines. Upper panel represents *TEAD4* motif sequence. Bottom panel represents the genes that overlap between the two cell lines. **C.** Correlation of expression and methylation of *TEAD4* enriched regions. Scatter plot shows the correlation between methylation levels at the *TEAD4* binding region and expression of its closest gene. The red dot represents significant inverse correlation between methylation levels at the *TEAD4* binding regions and gene expression in two cell lines (|fc| >= 2 and p-value < 0.05).

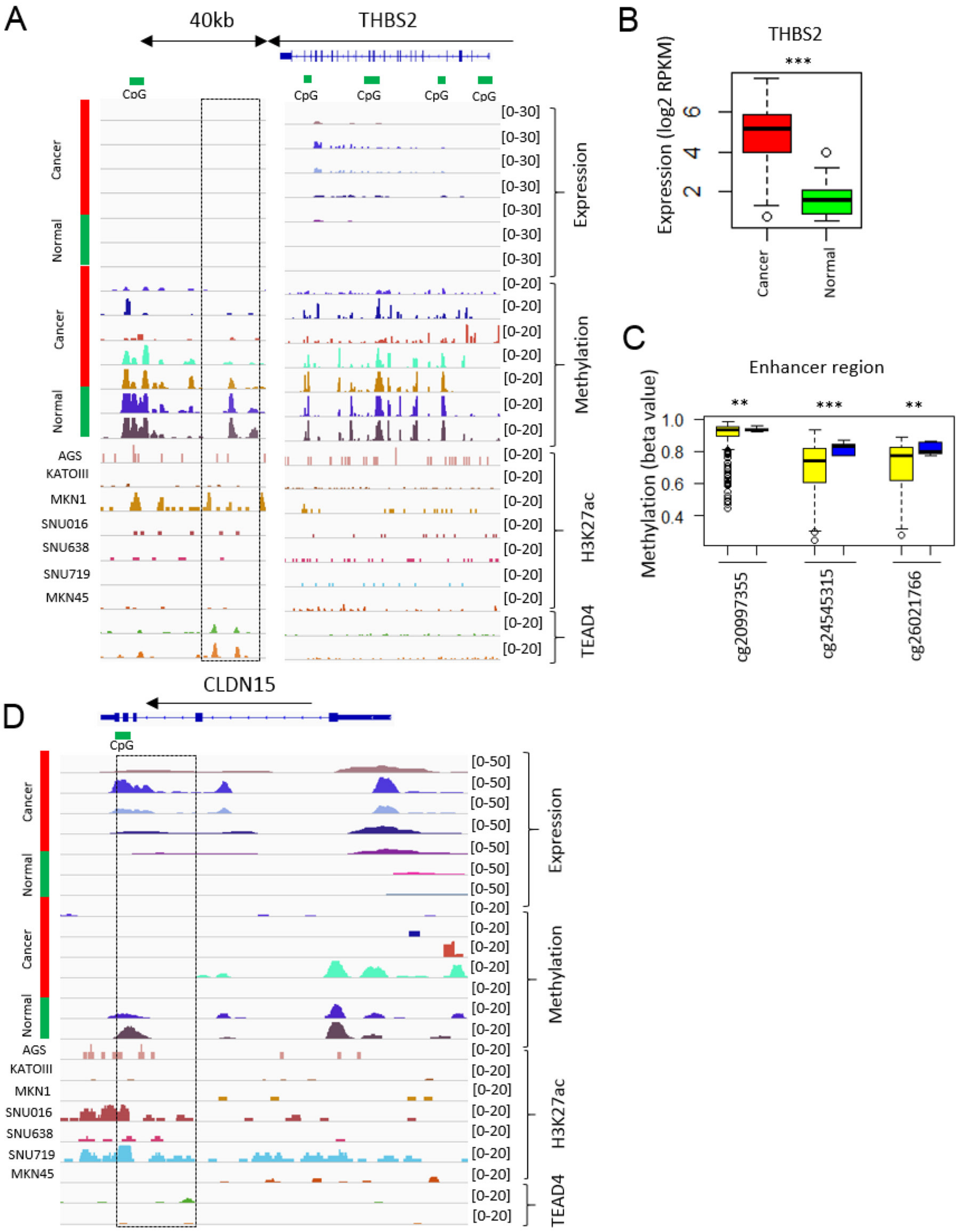

Supplementary Figure S2: Target genes of the hypo-methylated enhancers. A. The IGV screen shot shows methylation state at the distal enhancer region and expression of *THBS2* in gastric cancer. Bottom of track shows H3K27ac and *TEAD4* peaks at the *THBS2* enhancer locus. **B.** Expression level of *THBS2*. A box plot shows the expression level of *THBS2* in 212 gastric cancers compared with 28 normal samples. The red color represents cancer tissue and the green color represents normal tissue (*: p-value < 0.05; **: p-value < 0.005; ***: p-value < 0.0005). **C.** Methylation level of the THBS2 enhancer region. A box plot shows the methylation level of each probe at *THBS2* enhancer sites that contain three probes using the HumanMethylation450 BeadChip. The yellow color represents cancer tissue and the blue color represents normal tissue (*: p-value < 0.05; **: p-value < 0.005; ***: p-value < 0.0005). **D.** A screenshot of the IGV genome browser shows the intronic enhancer region of the *CLDN15* gene. Displayed tracks include the Chip-seq data for H3K27ac and TEAD4, MBD-seq, and mRNA-seq.

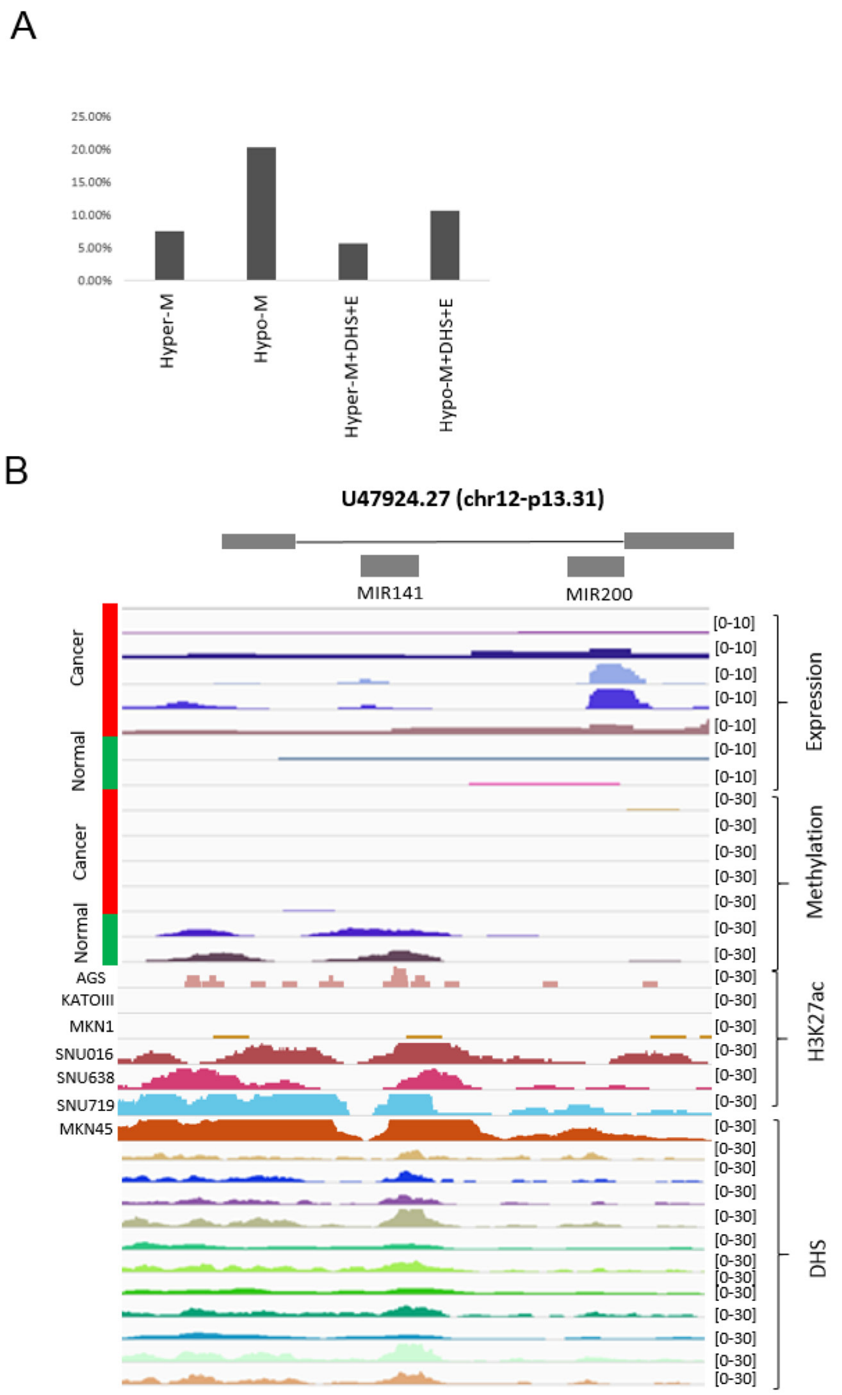

Supplementary Figure S3: Target lncRNAs of the hypo-methylated regions. A. Proportion of the overlap regions between the lncRNAs and regulatory regions. The x-axis represents differentially methylated regions such as Hyper-M (Hyper-methylation), Hypo-M (Hypo-methylation), Hyper-M+DHS+E (overlap regions among hyper-methylation, DHS, and enhancer regions), and Hypo-M+DHS+E (overlap regions among hypo-methylation, DHS, and enhancer regions). Y-axis represents percentage of the intersected regions between the lncRNAs and the differentially methylated region. **B.** A screenshot of the hypo-methylated and up-regulated lncRNA (U47924.27). The screen shot from IGV shows the methylation and expression levels of U47924.27 (Gencode v.19) in gastric cancer. Bottom track shows H3K27ac and DHS peaks in the U47924.27 locus.

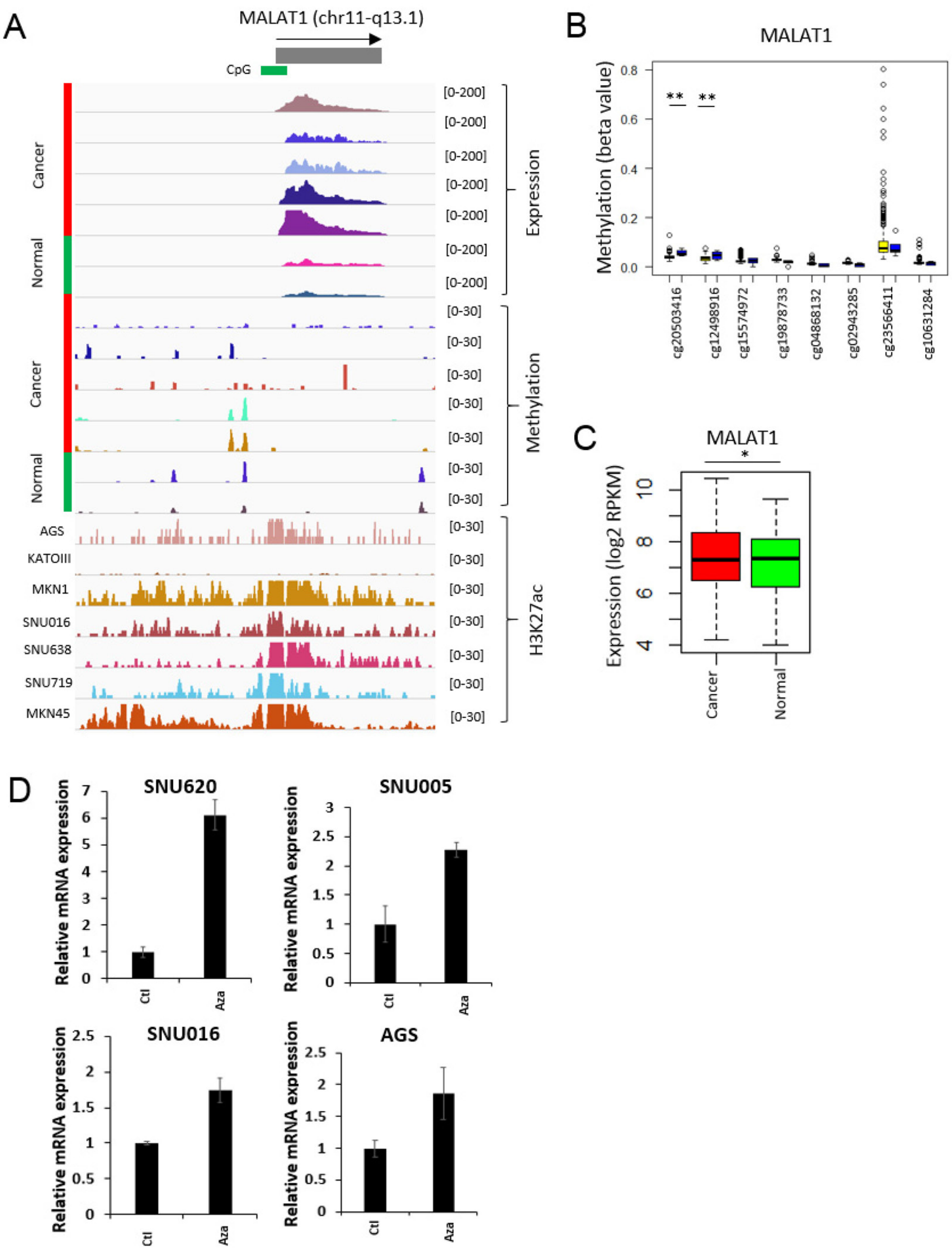

Supplementary Figure S4: *MALAT1* as a hypo-methylated lncRNA target. A. The screen shot from IGV illustrates the hypo-methylated and up-regulated lncRNA (*MALAT1*). Upper track shows the status of methylation and expression across gastric cancer and normal controls at the *MALAT1* locus. Bottom track shows H3K27ac peaks across gastric cancer cell lines. Red bar represents cancer tissues and green bar represents normal tissues. The dash dot line represents the hypo-methylated region of *MALAT1*. **B.** Methylation levels in the *MALAT1* promoter regions. The box plot shows the methylation level of *MALAT1* promoter that contains eight probes using the HumanMethylation450 BeadChip. Two probes are significantly hypo-methylated in gastric cancer compared with normal (cg20503416, and cg12498916; *: p-value < 0.05; **: p-value < 0.005; ***: p-value < 0.0005). The yellow box represents cancer samples and the blue box represents normal samples. **C.** A box plot shows the expression level of *MALAT1* in 240 gastric cancers compared with 28 normal samples (*: p-value < 0.05; **: p-value < 0.005; ***: p-value < 0.0005). **D.** *MALAT1* up-regulation by 5-Aza-dC in gastric cancer cell lines (SNU620, SNU005, SNU016, and AGS). Quantitative RT-PCR was performed using primers for *MALAT1*.

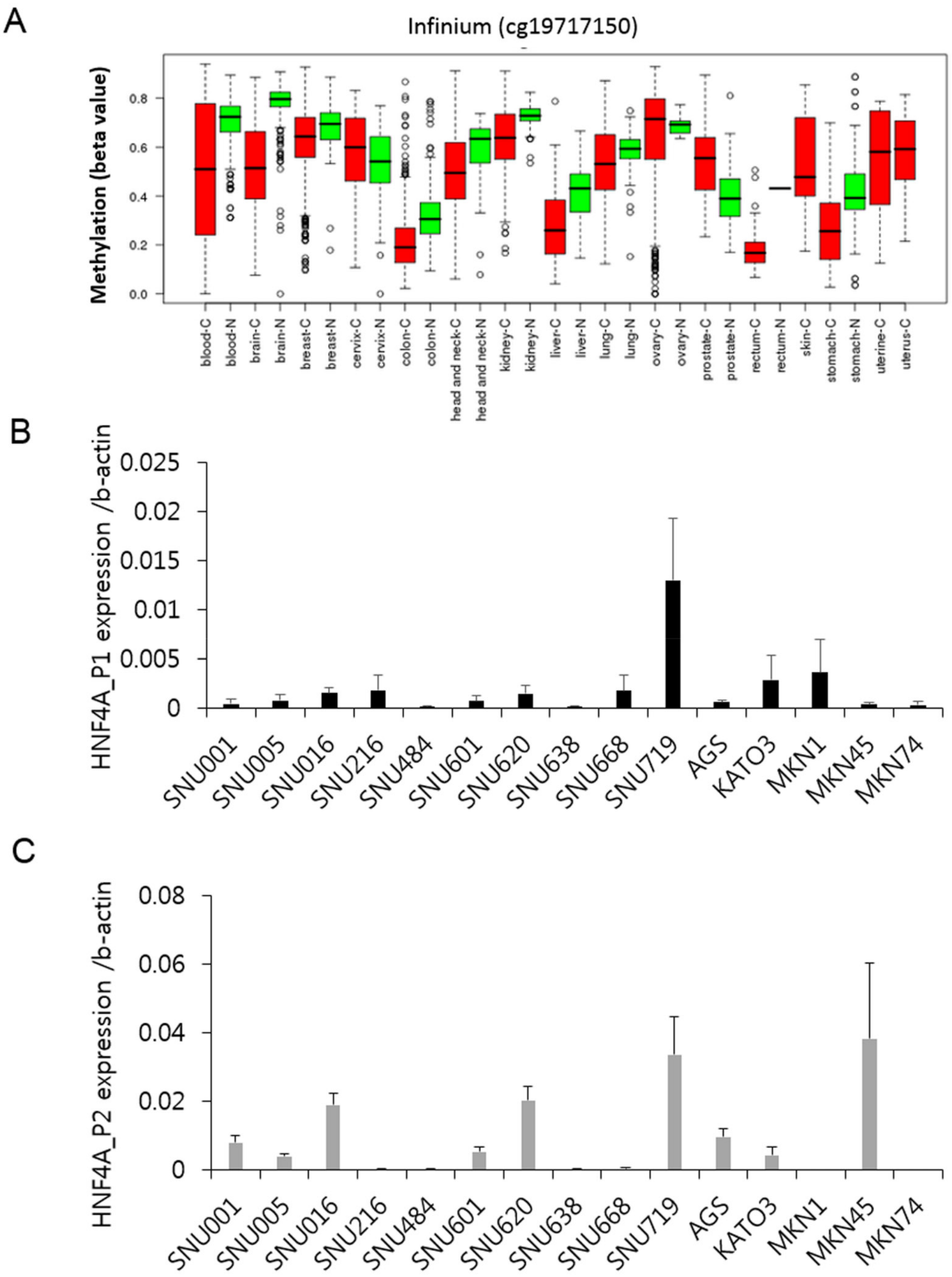

Supplementary Figure S5: Validation of the methylation and expression levels in *HNF4A*. A. Methylation pattern of *HNF4A* across diverse normal and tumor tissues from the MENT database (Infinium HumanMethylation27 BeadChip array). The red box represents cancer samples and the green box represents normal samples. **B.** Quantitative RT-PCR for HNF4A-P1 in 15 gastric cancer cell lines. **C.** Quantitative RT-PCR for HNF4A-P2 in 15 gastric cancer cell lines.

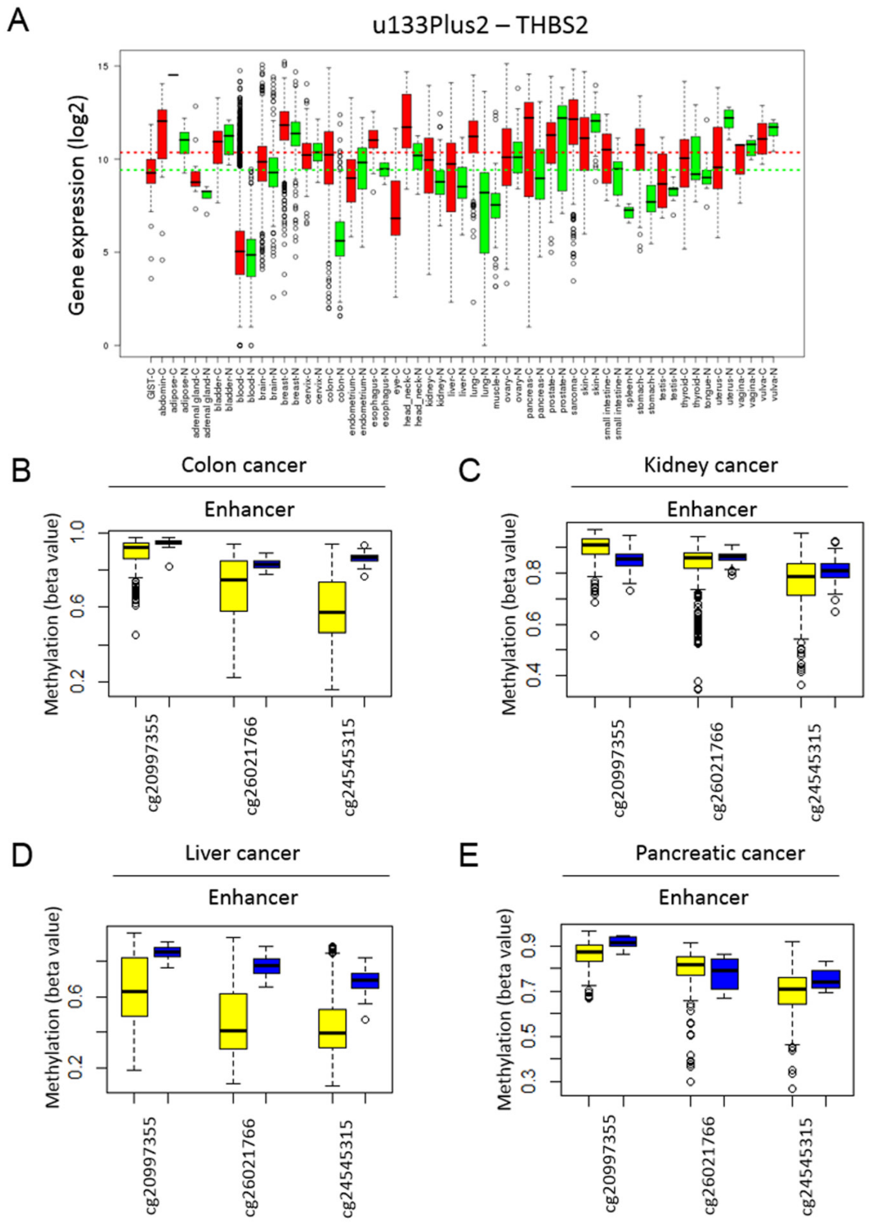

Supplementary Figure S6: The methylation and expression levels of *THBS2* in various cancers. A. Expression pattern of *THBS2* across diverse normal and tumor tissues using The GENT database (Human Genome U133 Plus 2.0 Array). The red box represents cancer samples and the green box represents normal samples. **B, C, D, E.** The box plot shows the methylation level of each probe at *THBS2* enhancer sites that contain three probes in colon cancer (B), kidney cancer (C), liver cancer (D), and pancreatic cancer (E) using the HumanMethylation450 BeadChip. The yellow color represents cancer tissue and the blue color represents normal tissue.

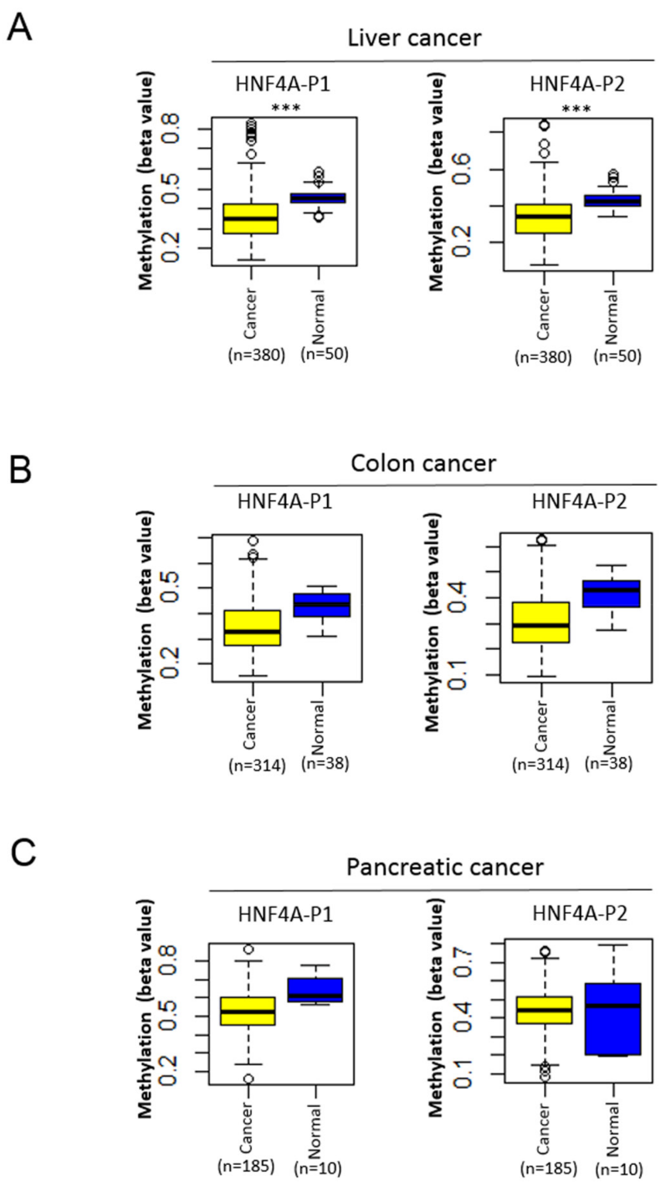

Supplementary Figure S7: The methylation levels of HNF4A promoters in three cancers. A. The boxplot shows the methylation levels of HNF4A-P1 and HNF4A-P2 promoters in liver cancer samples from the TCGA cohorts (HumanMethylation450k; n=430). **B.** The boxplot shows the methylation levels of HNF4A-P1 and HNF4A-P2 promoters in colon cancer samples from the TCGA cohorts (HumanMethylation450k; n=352). **C.** The boxplot shows the methylation levels of HNF4A-P1 and HNF4A-P2 promoters in pancreatic cancer samples from the TCGA cohorts (HumanMethylation450k; n=195).

Supplementary Table S1: Sample characteristics

	sex	age	Lauren Classification	T stage	N stage	Site	Vascular invasion	LN Number	Distant Meta
**CSC1**	F	69	Intestinal type	T3	N0	LC/UB	0	0/29	0
**CSC2**	M	79	Intestinal type	T4b	N3a	LC/LB	1	10/37	1
**CSC3**	M	51	Diffuse type	T4b	N0	LC/LB	1	0/66	0

Supplementary Table S2: Gene ontology of down-regulated genes hyper-methylated in the promoter

Term	P Value	Genes	Bonferroni	Benjamini	FDR
MAPK signaling pathway	0.0026	FGFR1, RPS6KA6, BDNF, RASGRF2, RASGRF1, NTRK2, PRKACB, FGF2	0.1515	0.1515	2.6740
Basal cell carcinoma	0.0071	WNT5A, LEF1, HHIP, GLI3	0.3554	0.1971	6.9852
Hedgehog signaling pathway	0.0075	WNT5A, HHIP, PRKACB, GLI3	0.3698	0.1426	7.3317
Neurotrophin signaling pathway	0.0119	RPS6KA6, BDNF, NTRK2, NGFRAP1, IRS1	0.5196	0.1674	11.3861
Insulin signaling pathway	0.0738	PYGL, PRKAR1A, PRKACB, IRS1	0.9907	0.6080	53.792
Pathways in cancer	0.0866	WNT5A, FGFR1, LEF1, HHIP, FGF2, GLI3	0.9960	0.6022	59.8239
Long-term potentiation	0.0870	RPS6KA6, GRIN2A, PRKACB	0.9961	0.5476	59.9707
Wnt signaling pathway	0.0958	WNT5A, PRICKLE1, LEF1, PRKACB	0.9978	0.5362	63.7093

Supplementary Table S3: Gene ontology of up-regulated genes hypo-methylated in the promoter

Term	P Value	Genes	Bonferroni	Benjamini	FDR
Maturity onset diabetes of the young	5.68E-04	HNF1A, HNF4A, FOXA2, MNX1	0.0280	0.02801	0.5459
Neuroactive ligand-receptor interaction	0.0891	GABRA2, DRD1, GABRR1, GABRB3, ADRA2A	0.9906	0.9032	59.331

Supplementary Table S4: Sample information of DNase I hypersensitive site data

Data	GSE	GSM	Tissue	platform	data download
DHS	GSE18927	GSM701498	Fetal Stomach	Illumina Genome Analyzer IIx	GEO
DHS	GSE18927	GSM701521	Fetal Stomach	Illumina Genome Analyzer IIx	GEO
DHS	GSE18927	GSM701528	Fetal Stomach	Illumina Genome Analyzer IIx	GEO
DHS	GSE18927	GSM701538	Fetal Stomach	Illumina Genome Analyzer IIx	GEO
DHS	GSE18927	GSM774202	Fetal Stomach	Illumina Genome Analyzer IIx	GEO
DHS	GSE18927	GSM774212	Fetal Stomach	Illumina Genome Analyzer IIx	GEO
DHS	GSE18927	GSM774232	Fetal Stomach	Illumina Genome Analyzer IIx	GEO
DHS	GSE18927	GSM817173	Fetal Stomach	Illumina Genome Analyzer IIx	GEO
DHS	GSE18927	GSM817199	Fetal Stomach	Illumina Genome Analyzer IIx	GEO
DHS	GSE18927	GSM878660	Fetal Stomach	Illumina Genome Analyzer IIx	GEO
DHS	GSE18927	GSM878665	Fetal Stomach	Illumina Genome Analyzer IIx	GEO

Supplementary Table S5: Gene ontology of up-regulated genes hypo-methylated in the enhancer

Term	P Value	Genes	Bonferroni	Benjamini	FDR
Axon guidance	0.0220	ABLIM2, SEMA5B, SEMA6A, ROBO1, EFNA2, EFNB1, NFATC2, NFATC1	0.8919	0.8919	21.8858
Focal adhesion	0.0306	VAV3, CCND2, COL6A2, COL6A1, COL1A1, THBS2, SHC2, COL5A1, PARVB, MYL9	0.9554	0.7890	29.2070
ECM-receptor interaction	0.0357	GP6, COL6A2, COL6A1, COL1A1, THBS2, COL5A1	0.9738	0.7030	33.2535
VEGF signaling pathway	0.0804	PLA2G10, NOS3, NFATC2, SHC2, NFATC1	0.9997	0.8770	60.5639
Calcium signaling pathway	0.0889	CHRM3, ATP2A3, CACNA1H, RYR2, NOS3, CHRNA7, CHRFAM7A, PLCB1, CACNA1C	0.999	0.8446	64.4256
Cardiac muscle contraction	0.0899	RYR2, COX6B2, TNNI3, CACNA1C, CACNA2D4	0.9999	0.7919	64.8533

Supplementary Table S6: Gene ontology of TEAD4 enriched peaks at enhancer hypo-methylated regions

Term	Genes	P-Value	Benjamini
Cell adhesion molecules (CAMs)	CDH1, CDH3, CLDN15, CLDN4, CLDN9, ITGB1, PVR	5.50E-03	3.60E-01
Pathways in cancer	KITLG, CDH1, EGLN3, EPAS1, ITGB1, RARA, RXRA, SLC2A1, TCF7 L2	4.80E-02	8.60E-01
Thyroid cancer	CDH1, RXRA, TCF7 L2	5.00E-02	7.50E-01
Leukocyte transendothelial migration	CLDN15, CLDN4, CLDN9, ITGB1, VAV2	5.80E-02	7.00E-01

Supplementary Table S7: List of gastric samples from Readmap Epigenomics Project

EID[1)]	Epigenome name	state
E084	Fetal intestine large	6_EnhG[2)], and 7_Enh[3)]
E085	Fetal intestine small	6_EnhG[2)], and 7_Enh[3)]
E092	Fetal stomach	6_EnhG[2)], and 7_Enh[3)]
E094	Gastric	6_EnhG[2)], and 7_Enh[3)]
E109	Small intestine	6_EnhG[2)], and 7_Enh[3)]
E110	Stomach mucosa	6_EnhG[2)], and 7_Enh[3)]

1)EID-reference epigenome identifier
2)6_EnhG-Genic enhancers
3)7_Enh-Enhancers
*Detailed chromatin state http://www.nature.com/nature/journal/v518/n7539/extref/nature14248s1.pdf

Supplementary Data S1: Differentially methylated regions (DMRs) in gastric cancer compared to normal

See Supplementary File 1

Supplementary Data S2: list of over-expressed genes by hypomethylation at repeat region

See Supplementary File 2

Supplementary Data S3: Gene list as methylation targets at promoter regions

See Supplementary File 3

Supplementary Data S4: Gene lists as methylation targets at enhancer regions

See Supplementary File 4

Supplementary Data S5: Intersected DMRs between gastric specific enhancers and hyper-methylated regions in GC compared to normal

See Supplementary File 5

Supplementary Data S6: LncRNA lists as methylation targets at promoter region of lncRNAs

See Supplementary File 6

Supplementary Data S7: Sample information of subtype-specific methylation analysis

See Supplementary File 7

Supplementary Data S8: Gene lists as subtype-specific methylation target

See Supplementary File 8
